# Editorial: Epidemiology of the transboundary swine diseases in Asia & Pacific

**DOI:** 10.3389/fvets.2024.1383900

**Published:** 2024-02-20

**Authors:** Satoshi Ito, Jaime Bosch, Norikazu Isoda, Lam Thanh Nguyen, Marta Martinez Aviles

**Affiliations:** ^1^South Kyushu Livestock Veterinary Medicine Center, Kagoshima University, Soo, Japan; ^2^VISAVET Health Surveillance Centre, Complutense University of Madrid, Madrid, Spain; ^3^Department of Animal Health, Faculty of Veterinary Medicine, Complutense University of Madrid, Madrid, Spain; ^4^Laboratory of Microbiology, Department of Disease Control, Faculty of Veterinary Medicine, Hokkaido University, Sapporo, Japan; ^5^Global Station for Zoonosis Control, Global Institute for Collaborative Research and Education, Hokkaido University, Sapporo, Japan; ^6^Faculty of Veterinary Medicine, College of Agriculture, Can Tho University, Can Tho, Vietnam; ^7^Centro de Investigación en Sanidad Animal (Animal Health Research Centre), CISA-INIA/CSIC, Madrid, Spain

**Keywords:** transboundary diseases, Asia-Pacific, epidemiology, pig, wild boar, ASF, CSF, PRRS

Asia is a leading market for pork production and consumption, contributing to over half of the world's pork supply. Smallholder-based swine management systems, which are sensitive to infectious diseases, are still prevalent in many Asian countries and directly linked to the vulnerability of the pig value chain at the national level. This is a major characteristic of the swine production in many Asian countries. For these traditional pig farming systems, the industry is now facing a critical turning point. In recent years, several transboundary swine diseases such as African Swine Fever (ASF), Classical Swine Fever, Foot and Mouth Disease, and Porcine Reproductive and Respiratory Syndrome (PRRS) have been reported across Asia, posing a potential catastrophic impact on swine production and raising significant concerns.

As evident from our Research Topic, in which 70% of the published papers are related to ASF research, ASF has received significant attention in Asia. Since its first detection in China in 2018, ASF has continued to have a severe impact on pig farming across Asia. Ito, Kawaguchi et al. conducted a comprehensive epidemiological analysis based on existing literature data and publicly available open databases to understand the ASF epidemic in Asia. Although there has been a decline in the official reports of ASF recently, this trend suggests not so much an end of the epidemic, but rather its possible endemicity in the region. As a first step toward control under these circumstances, identifying the overall pattern of the epidemic and the risk factors for its spread are critical.

Lee et al. conducted a spatiotemporal analysis and assessment of potential risk factors along the pig value chain in Lao Cai province, Vietnam. They identified spatiotemporal clusters and potential risk factors attributed to geographical features. Farmers recognized a high ASF transmission risk from visitors to farms, highlighting the importance of biosecurity across the entire pig value chain. In Oudomxay province, northern Laos, a case study of smallholder pig farming systems was conducted by Matsumoto et al.. The study investigated the management of pigs in villages affected by ASF and analyzed the frequency of risk factors and stock losses at the household level. It identified swill feeding and free-ranging as risk factors for ASF, as well as inadequate biosecurity measures leading to contamination of the environment. These studies emphasize the need for improved knowledge, awareness, and understanding of ASF infection and risk at the community level, along with the need for enhanced disease management resources from local to governmental levels.

On individual farm scales, the development and swift implementation of evidence-based measures are crucial. Understanding the mechanisms of virus transmission plays a significant role as the first step in infectious disease control strategies at the site level. Li et al. reported for the first time the evidence of ASF virus (ASFV) aerosol transmission under field conditions, previously documented only in experimental settings. In general, a time lag exists between infection occurrence and detection on farms, which significantly influences the scale of outbreak spread. Yoon et al. estimated a median interval of 9.0 days (Q1–Q3, 7.8–10.5 days) from infection to detection in South Korean ASF outbreak farms, noting variations by breeding stage and farm type. This finding is essential for early control of infectious disease outbreaks. In infected farms, culling all animals is a standard containment strategy, but challenges such as lack of human resources and inadequate facilities can hinder its execution. In Vietnam, where continuous large-scale outbreaks occur, a spot removal approach—rapidly identifying and eliminating infected individuals considering economic situation—is permitted. While this method reduces the economic burden on farmers, it risks missing potentially infected individuals. Mai et al. evaluated the effectiveness of this approach by calculating the basic reproduction number (R_0_) for the in-farm spread of ASF in two midsize commercial pig farms in Vietnam.

The unprecedented outbreak of ASF underscores the need for fundamental changes in Asia's traditional pig farming management systems and may indirectly contribute to the prevention of other diseases. Zhao et al. conducted risk factors and spatiotemporal analysis of PRRS seroprevalence in China before and after the ASF outbreak, finding that the likelihood of farms being PRRSV antibody-positive was 3.1 times higher before the ASF outbreak, likely due to enhanced biosecurity measures post-ASF. Similarly, Fan et al. revealed the prevalence of Porcine Circovirus type 2 (PCV2) across China, a virus classified as an emerging infectious disease causing significant economic losses in the global pig industry. The study found variability in PCV2 positivity rates based on farm type and breeding stages, along with regional differences. These patterns suggest that the variations in protocols implemented since 2018 for ASF containment might have influenced these trends.

The recent ASF outbreaks have significantly impacted the swine industry in Asia, yet it is crucial not to overlook the severe damage caused by other swine diseases, such as PRRS and PCV2 mentioned earlier. The emergence of the Porcine Deltacorona virus (PDCoV), spreading globally with notable outbreaks in Asian countries, raises widespread concerns. Through systematic review and meta-analysis, Sun et al. calculated the estimated prevalence of PDCoV infection in pig populations in mainland China, revealing a high prevalence of 12.4%, highlighting the urgent need for enhanced biosecurity prevention and control measures.

Research on swine diseases in Asia predominantly targets domestic populations. However, the potential contribution of wildlife to disease transmission and maintenance deserves attention. Thoroughly elucidating this role is critical for holistic disease management strategies. In Asia, where wildlife surveillance systems are not fully established, further research in this field is also encouraged.

As emphasized by Ito, Bosch et al., the risk of global spread of the lower virulent ASFV reported in China highlights a scenario where Asia's current challenges could escalate into a worldwide problem. Asia stands as a major hub for swine production, with strong connections to countries worldwide. To prevent potential worldwide infectious disease outbreaks, continuous monitoring of the livestock industry within the Asia-Pacific region is essential.

## Author contributions

SI: Conceptualization, Writing—original draft, Writing—review & editing. JB: Writing—review & editing. NI: Writing—review & editing. LT: Writing—review & editing. MM: Writing—review & editing.

